# Examining the Resilience of University Students: A Comparative Mental Health Study

**DOI:** 10.7759/cureus.69293

**Published:** 2024-09-12

**Authors:** Aina Farahim Suhaimi, Nurulumi Ahmad, Habibah Kamaruzaman

**Affiliations:** 1 Clinical Pharmacy, Faculty of Pharmacy, Universiti Sultan Zainal Abidin, Terengganu, MYS

**Keywords:** anxiety, depression, mental health, resilience, stress, university students

## Abstract

Background: This cross-sectional study aimed to investigate the resilience levels and mental health outcomes among university students, specifically those in the science and non-science fields. Mental well-being varies widely among individuals, particularly among university students. Thus, the aim of this study was to examine and compare the level of depression, anxiety, and stress betweenundergraduate students of the Faculty of Bio-Resources and Food Industry (FBIM) and Faculty of Social Sciences and Humanities (FSSG) from Universiti Sultan Zainal Abidin (UniSZA), Terengganu, Malaysia, as well as to identify the correlations between resilience and their mental health status.

Materials and methods: This study was conducted among FBIM and FSSG students of UniSZA starting from March 18, 2024, to May 31, 2024. A total of 329 students were assessed for this purpose. All participants answered questionnaires that were prepared based on the Depression, Anxiety, and Stress Scale-21 (DASS-21) and the Connor and Davidson Resilience Scale (CD-RISC) through a Google Form (Google Inc., Mountain View, CA).

Results: The Pearson's chi-square test, Mann-Whitney U test, and Kruskal-Wallis H test were used to analyze the data in this study. This study showed that the students from FBIM had poorer mental health than those from the FSSG (p<0.001). The results revealed statistically significant differences between the levels of depression, anxiety, and stress between students of the two faculties. Moreover, the research correlated the level of depression, anxiety, and stress with the resilience level to examine how individuals cope with psychological challenges.

Conclusion: This outcome might be connected to students' resilience to overcome mental health issues. This research could potentially assist students in identifying their mental health status, as some may not be aware of their underlying brain conditions.

## Introduction

The World Health Organization (WHO) defines mental health as the inner balance that enables individuals to overcome problems, reach their potential, excel academically and professionally, and contribute to society [1]. Energy and harmony enhance our ability to make decisions, connect, and shape our environment. Mental health is a fundamental right for all. It is crucial for personal growth, community well-being, and socioeconomic advancement [1]. Health has traditionally been defined as a condition of complete physical and mental well-being, free from illness, suffering, or incapacity [2]. Every day, college students encounter several problems that can affect their mental health. Due to particular obstacles and pressures, university students have a higher risk of mental health issues compared to the general population [2].

Effective approaches to increasing emotional well-being require a strong understanding of resilience and the ability to overcome obstacles and suffering. Mental health depends on resilience, which is defined as a feature that distinguishes individuals and aids in stress management [3]. Today, resilience encompasses the ability to overcome challenges and thrive. Research emphasizes the significance of mental health [3]. It is crucial for adapting to the academic environment as well as for university success. To say that individuals are resilient, they exhibit the unique ability to overcome adversities and find a balance [4]. Another study defines resilience as the ability to overcome stress and thrive despite challenges. Research shows that resilience leads to better mental health. Resilience empowers individuals to overcome challenges and hurdles in life [5]. According to Connor and Davidson, resilience is the inner strength that helps individuals overcome adversity and thrive [6].

Faculty of Bio-Resources and Food Industry (FBIM) and Faculty of Social Sciences and Humanities (FSSG) were chosen to represent science and non-science fields, respectively. The differences in attitudes towards mental health and resilience between science and non-science faculty can be significant. A study found that science faculty members often exhibit more negative attitudes toward inclusive teaching strategies compared to their counterparts in the humanities, which may reflect broader cultural attitudes toward mental health and emotional expression in these disciplines [7]. This disparity can influence how faculty members approach their own mental health and the support they provide to students, highlighting the need for tailored interventions that consider disciplinary differences.

Resilience entails adapting to changes, taking responsibility, and overcoming challenges and unpredictability, including both positive and negative situations. This quality showcases the evolution of innovation and adaptability over time. A study by Wagnild indicates resilience was identified as a valuable personal asset, comprising positive attributes that empower individuals and aid in adapting to challenges and life transitions [8]. Research indicates that resilience can improve well-being and life satisfaction [9]. Both direct and indirect links exist between resilience and well-being. Resilience enables individuals to handle and adapt to difficult situations, promoting equilibrium. It promotes successful adaptive coping techniques. Research by Innes et al. and Tugade et al. indicates that effective action in stressful settings is enhanced [10, 11]. Problem-focused solutions have been found to improve mental well-being. Research suggests that techniques like ignoring emotions, diverting, minimizing situations, seeking meaning, blaming oneself, and expressing sentiments are associated with lower resilience, life satisfaction, and positive emotions. However, these tactics are associated with an increase in mental health issues [12, 13]. Being resilient significantly affects the emotional well-being of college students. Research indicates that resilient kids have greater mental health and reduced stress levels. Meanwhile, individuals with resilience are likely to have higher academic performance and personal happiness compared to those lacking resilience. To successfully support students, educational institutions should offer specialized programming and interventions that enhance resilience. It is important to consider elements that may affect resilience in different student populations.

This study examined the association between resilience and mental health markers among university students and investigated the relationship between resilience and both negative and positive markers, such as stress and life satisfaction.

## Materials and methods

The cross-sectional study was undertaken at Universiti Sultan Zainal Abidin (UniSZA) in Terengganu. To ensure the study's credibility, the participants were current university students. Undergraduate students from the FBIM and the FSSG who participated in this study were chosen to represent the science and non-science fields, respectively. A six-person pilot study was done and indicated that the participants comprehended the questions well. There were no significant issues identified with the clarity or structure of the questionnaire.

The data were collected from March 18 to May 31, 2024. The two sections of questionnaires were adapted, consisting of the Connor and Davidson Resilience Scale (CD-RISC) [6] (Appendix A) and the Depression, Anxiety, and Stress Scale-21 (DASS-21) (Appendix B) [9], and were distributed using Google Forms (Google Inc., Mountain View, CA). Lecturers and student representatives from each faculty were contacted to facilitate the distribution process. Participation was on a voluntary basis. These surveys aimed to study mental health concerns by measuring depression, anxiety, and stress levels with the resilience level in the face of adversity between these students, as approved by the UniSZA Human Research and Ethics Committee (UHREC) (approval number: UniSZA/UHREC/2024/616).

Recruited participants were informed about research topics and potential risks, as they were crucial to reducing dropout rates. Informed permission was obtained from all the participants before the experiment began. The confidentiality of all personal data collected from the participant was strictly maintained as stated in the Google form. The sample size was calculated using the Raosoft online sample calculator (Raosoft Inc., Seattle, WA), maintaining a 95% confidence level and a 5% error margin. A population proportion of 50% and a Z value of 1.96 were used [13]. The study required a minimum of 320 respondents from these faculties.

Using the IBM SPSS Statistics software for Windows, Version 25.0 (IBM Corp., Armonk, NY), the data were analyzed according to the demographic variables in a descriptive manner. Once the data were gathered, they were organized and displayed using tables, graphs, and charts. In this study, we examined the correlation between depression, anxiety, and stress and their resilience levels regarding their experience in university life. These relationships were evaluated using statistical methods, specifically utilizing Pearson’s chi-square test to compare the association between the categorical variables in depression, anxiety, and stress levels in relation to resilience among FBIM and FSSG students, male and female students, and students aged 20 and 21. A statistical significance threshold of p<0.05 was selected. Meanwhile, the Mann-Whitney U test and the Kruskal-Wallis H test were used for non-parametric data in this study. The data were analyzed to compute the mean scores for depression, anxiety, stress, and resilience levels. This was done using responses to multiple-choice questions (MCQ) that are pertinent to these factors. For the DASS-21, a higher score may indicate a greater prevalence of mental health issues among university students, while a lower score suggests a lower prevalence. In contrast, the CD-RISC reflects resilience levels, where higher scores represent stronger resilience and lower scores indicate reduced resilience.

## Results

The respondents, who were pursuing undergraduate degrees, ranged in age from 18 to 25 years. One hundred and twenty-six males and 203 females made up this group, with 171 hailing from the FBIM and 158 from the FSSG. Of these, 52% were enrolled in the FBIM, while 48% were in the FSSG, with 38.3% of them being male and 61.7% being female, which varied in age. The baseline characteristics of the participants are detailed in Table 1. Table 2 illustrates the frequency of depression, anxiety, and stress for both faculties according to the DASS-21 score.

**Table 1:** Demographic data of the study participants FBIM: Faculty of Bio-Resources and Food Industry; FSSG: Faculty of Social Sciences and Humanities

Variable	Classification	Frequency	Percentage (%)
Faculty	FBIM	171	52.0
	FSSG	159	48.0
Gender	Male	126	38.3
	Female	203	61.7
Age (in years)	18	14	4.3
	19	29	8.8
	20	51	15.5
	21	102	31.0
	22	78	23.7
	23	43	13.1
	24	8	2.4
	25	4	1.2

**Table 2:** Frequency of depression, anxiety, and stress N: number of students; DASS-21: Depression, Anxiety, and Stress Scale-21

Level of mental status	Depression			Anxiety			Stress		
	N	%	DASS-21 Score	N	%	DASS-21 Score	N	%	DASS-21 Score
Normal	0	0.0	0 – 4	0	0.0	0 – 3	25	7.6	0 – 7
Mild	0	0.0	5 – 6	0	0.0	4 – 5	55	16.7	8 – 9
Moderate	119	36.2	7 – 10	27	8.2	6 – 7	63	19.1	10 – 12
Severe	58	17.6	11 – 13	64	19.5	8 – 9	87	26.4	13 – 16
Extremely severe	152	46.2	14+	238	72.3	10+	99	30.1	17+

As shown in Table 3, the FBIM group had a higher mean depression score (mean = 4.57, n = 171, CI = 4.46-4.68) than the FSSG group (mean = 3.59, n = 158, CI = 3.46-3.71). The FBIM students had a higher anxiety score than the FSSG participants (mean = 4.91, n = 171, CI = 4.86-4.96). Since one group was not normally distributed, a non-parametric test was chosen. The FBIM participants had higher stress levels than the FSSG participants (mean = 4.25, n = 171, CI = 4.11-4.39). Both groups had pretty normal stress distributions that were near zero. Therefore, a parametric test was conducted for these variables.

**Table 3:** Depression, anxiety, and stress in different faculties N: number of students; %: percentage; SD: standard deviation; FBIM: Faculty of Bio-Resources and Food Industry; FSSG: Faculty of Social Sciences and Humanities; p-value<0.05 (significant)

Variables			Pearson's chi-square test	Mann-Whitney U test	p-value
		N / %	Mean ± SD	Mean Rank	p-value
Depression	FBIM	171 (52%)	4.57 ± 0.22	-	p<0.001
	FSSG	158 (48%)	3.59 ± 0.26	-	
Stress	FBIM	171 (52%)	4.25 ± 0.28	-	p<0.001
	FSSG	158 (48%)	2.78 ± 0.27	-	
Anxiety	FBIM	171 (52%)	-	197.61	p<0.001
	FSSG	158 (48%)	-	129.71	

As shown in Table 3, Pearson's chi-square test displays significant depression differences between FBIM and FSSG students (p<0.001), with a mean FBIM score of 4.57 and a mean FSSG score of 3.59. In other words, FBIM students have a significantly higher depression score than FSSG students. Apart from that, FBIM and FSSG students had significantly different stress levels (p<0.001), with FBIM at 4.25 and FSSG at 2.78. This means FBIM students experienced more stress than FSSG students. Non-parametric Mann-Whitney U tests measured the anxiety levels of FBIM and FSSG students. According to the result, FBIM and FSSG students had significantly different anxiety levels (p<0.001), with mean ranks of 197.61 and 129.71, respectively, according to the Mann-Whitney U test. Thus, FBIM students showed more anxiety than FSSG students. 

Male and female students displayed significantly different association depression levels (p<0.001), with males averaging 4.28 and girls 3.99, according to Pearson's chi-square test. Henceforth, these data revealed that male students have a significantly higher depression score than females. Besides that, male students had a mean anxiety score of 4.69, whereas female students had 4.61 (p<0.001). Therefore, male students not only had more anxiety than females but also significantly higher. The male students had a mean stress level of 3.74, whereas the female students had 3.43 (p<0.001). This shows that men had a significantly higher stress score than women (Table 4).

**Table 4:** Depression, anxiety, and stress in different genders N: number of students; %: percentage; SD: standard deviation; p-value<0.05 (significant)

Variables		Pearson's chi-square test (p-value)		
		N / %	Mean ± SD	
Depression	Male	126 (38%)	4.28 ± 0.31	p<0.001
	Female	203 (62%)	3.99 ± 0.42	
Anxiety	Male	126 (28%)	4.69 ± 0.22	p<0.001
	Female	203 (62%)	4.61 ± 0.18	
Stress	Male	126 (28%)	3.74 ± 0.46	p<0.001
	Female	203 (62%)	3.43 ± 0.34	

Pearson's chi-square test showed a significant association difference in depression between 21- and 20-year-old students (p<0.001), with a mean of 4.42 and 4.20, respectively. Student depression was higher among 21-year-olds than among 20-year-olds. The 21-year-old group had a far higher depression score than the 20-year-old group. This is because the mean stress level for 21-year-olds was 4.01, compared to 3.63 for 20-year-olds (p<0.001). Obviously, the 21-year-old group was far more stressed than the 20-year-old group. Besides that, the Kruskal-Wallis H test showed that 21-year-olds had a mean anxiety rank of 108.40 and 20-year-olds 103.54 (p<0.001). The mean anxiety level of 21-year-olds was higher than 20. Thus, the 21-year-old group had a higher anxiety score than the 20-year-old group (Table 5).

**Table 5:** Depression, anxiety, and stress in different age N: number of students; %: percentage; SD: standard deviation; p-value<0.05 (significant)

Variables			Pearson's chi-square test	Mann-Whitney U test	p-value
		N / %	Mean ± SD	Mean Rank	p-value
Depression	21-years old	102 (67%)	4.42 ± 0.39	-	p<0.001
	20- years old	51 (33%)	4.20 ± 0.49	-	
Stress	21-years old	102 (67%)	4.01 ± 0.46	-	p<0.001
	20-years old	51 (33%)	3.63 ± 0.67	-	
Anxiety	21-years old	102 (67%)	-	108.40	p<0.001
	20- years old	51 (33%)	-	103.54	

The DASS-21 and the CD-RISC had consistently correlated negatively. Research shows that CD-RISC resilience scores are linked to reduced DASS-21 subscales for depression, anxiety, and stress. In this study, depression, anxiety, and stress were negatively correlated with resilience (r = -0.454, -0.331, and -0.340) [13]. As shown in Table 6, the findings suggest resilience protects against negative psychological stress. Higher depression levels were associated with decreased resilience (r = -0.287), according to Pearson correlation. Anxiety was inversely connected with resilience (r = -0.206), indicating that higher anxiety levels impair resilience. Stress was also inversely correlated with resilience (r = -0.264), indicating that higher stress levels impair resilience. Resilience had a negative correlation with depression, anxiety, and stress, suggesting that it mitigates these mental health issues. All correlation coefficients exhibited poor performance, as they deviated significantly from one and approached 0 [14]. The probability of random variation in the sample was less than 1% for all relationships (p = 0.001). Hence, the strong negative connections between resilience and depression, anxiety, and stress showed that resilience may guard against these mental health issues.

**Table 6:** Correlation between resilience and depression, anxiety, and stress

Correlation coefficients (r)	Level of depression	Level of anxiety	Level of stress
Level of resilience	-0.287	-0.206	-0.264
p-value	0.001		

## Discussion

Mental health status in different faculties

This study examined the prevalence, relationships, and causes of depression, anxiety, and stress among students from the FBIM (science) and the FSSG (non-science) schools. This study found that FBIM students had higher levels of depression, anxiety, and stress than FSSG students. The statistical significance of this result was confirmed by comparing students from the two faculties (p<0.001). An Indian study found that science students have higher levels of depression, anxiety, and stress than non-science students [14]. More specifically, 3.86% of scientific students felt stress, 96.14% felt anxiety, and 88.46% felt depression. Stress was lower than depression and anxiety in a cross-sectional survey of university students from diverse countries. The mixed-methods study collected and analyzed data using quantitative and qualitative methods. Academic burnout was highest in sciences and lowest in non-science studies. Research consistently shows that science students have higher rates of depression, anxiety, and stress than non-science students. Another Indian survey found 62.6% of science students have depression, 67.8% anxiety, and 17.8% stress [15].

In this study, FBIM students exhibited a substantially higher mean depression level (mean = 4.57, CI = 4.46 to 4.68) than FSSG students (mean = 3.59, CI = 3.46 to 3.71). Pearson's chi-square test was selected based on the normal distribution data. FBIM students had significantly higher depression levels than FSSG students (p = 0.001). This significant difference highlights the need for FBIM student-specific mental health treatments to meet their unique challenges. Furthermore, FBIM students had higher anxiety levels than FSSG participants (mean = 4.91, CI = 4.86 to 4.96). However, the skewness and kurtosis values showed that FBIM students' anxiety data were not normal, while FSSG data were somewhat normal. Thus, the Mann-Whitney U test was chosen to compare anxiety levels. From this test, FBIM students showed a higher mean rank (197.61) than FSSG students (129.71) for anxiety level (p = 0.001). Because FBIM students had non-normal anxiety levels, alternative or supplemental support strategies that target anxiety concerns are necessary.

The FBIM students had significantly greater stress levels (mean = 4.25, CI = 4.11-4.39) than FSSG students (mean = 2.78, CI = 2.60-2.97). Both groups demonstrated normal stress distributions with skewness and kurtosis within the allowed range. For stress comparisons, Pearson's chi-square test was appropriate. The FBIM students had significantly higher stress levels than FSSG students (p = 0.001). This study suggests that FBIM students should prioritize stress management programs and resources to reduce their high-stress levels.

A cross-sectional study in Hyderabad, India, found that university students have high rates of depression, anxiety, and stress [16]. The science group, for instance, exhibited a higher mean level of anxiety, suggesting that students in this group may experience greater levels of worry and stress in comparison to students in non-science areas. The results of this study align with prior research that has emphasized the significance of providing mental health assistance to students in the science discipline. This study's findings emphasize the necessity of incorporating mental health support services into the curriculum and policies of educational institutions. This encompasses offering students access to counseling services, advocating for mental health awareness, and cultivating a nurturing and inclusive learning atmosphere. Educational institutions can also play a pivotal role in fostering mental well-being by offering students resources and assistance to effectively cope with stress and anxiety. The university could develop tailored interventions to address specific challenges in each faculty, such as targeted workshops, mentorship programs, or academic support systems. Besides that, universities could also introduce early screening programs using tools like DASS-21 to identify students who may be at risk of mental health issues. This can help provide early interventions, such as counseling services before problems escalate.

Mental health status in different genders

Based on the results, male college students have higher degrees of depression, anxiety, and stress than female students. Male students had a higher mean depression level (mean = 4.28, CI = 4.12-4.43) than female students (mean = 3.99, CI = 3.87-4.11). Men had more anxiety (mean = 4.69, CI = 4.58-4.80) than women (mean = 4.61, CI = 4.52-4.70). Male students were more stressed (mean = 3.74, CI = 3.51-3.97) than female students (mean = 3.43, CI = 3.26-3.60). Pearson's chi-square test showed statistical significance for the differences. These findings support other research on university student mental health inequities by gender. Gao et al., for example, found that more male students than girls have depression. Female students have increased anxiety in their first two years of university [17]. According to a UK study, male students are more likely to experience stress and mental health issues than female students [13]. Societal standards, coping strategies, and aid-seeking contribute to gender differences. Cultural expectations and gender stereotypes make male students more likely to engage in risky behavior and less likely to seek mental health help [18]. University life and its academic and social pressures may have a greater impact on male students' mental health [19]. These findings highlight the need for targeted mental health treatments and assistance for male university students. To address these inequalities, universities should consider gender-specific mental health programs, mental health literacy, and a welcoming campus environment [20]. We can also establish peer-led support groups that focus on mental health awareness and resilience-building. Students may feel more comfortable sharing their experiences with peers, which can lead to improved coping strategies and overall mental health outcomes.

Mental health status in different age groups

This study included 18 to 25-year-olds. However, the overall data only included 20- and 21-year-olds because they are more likely to have major mental health issues. University students aged 20 and 21 have a higher rate of major mental health problems than other age groups. The shift into senior years may make 20- and 21-year-old students more vulnerable, as most are in their second or third year of study. The 21-year-olds had much more depression, anxiety, and stress than the 20-year-olds. To illustrate this, the present study found that 21-year-old students were more depressed than 20-year-olds (mean = 4.42, CI = 4.25-4.59). The mean anxiety level was higher among 21-year-olds (mean = 4.81, CI = 4.72-4.91) than 20-year-olds (mean = 4.75, CI = 4.59-4.90). The mean stress level of 21-year-old students was higher than that of 20-year-olds (mean = 4.01, CI = 3.78-4.24). The findings support previous research suggesting mental health issues peak in the early 20s and that older students are more likely to experience depression, anxiety, and stress [19]. The data also suggest that university life and the combined academic and social duties may have a greater influence on older students, potentially affecting their mental health [19]. The study stresses the need for mental health treatments and assistance for university students, especially older students. To address these inequalities, universities may want to promote mental health literacy, age-specific activities, a welcoming campus, and also create interdisciplinary programs that encourage collaboration between science and non-science students. This may help reduce isolation and stress. Joint projects or study groups can foster a supportive learning environment.

Faculty differences in resilience levels

The FSSG students are more resilient than those in the FBIM. More FSSG students were high average and superiorly resilient, while more FBIM students were low average and average. These findings support previous research showing that social science and humanities students are more resilient than technical or scientific students. Academic programs in social science often emphasize interpersonal skills, critical thinking, and adaptability, which may explain this. These traits are crucial to resilience [20].

Gender differences in resilience levels

The study identified no significant resilience differences between male and female students. Both groups had equal resilience distributions, with a slightly higher number of male students in superior. These findings contradict previous findings that female students are more resilient [21]. This study found no significant differences between gender and resilience, suggesting that academic discipline and age may have a greater impact on resilience.

Age differences in resilience levels

The results indicate that 21-year-olds were more resilient than 20-year-olds. These data suggest that resilience increases with age as students gain life experiences and coping mechanisms to tackle academic challenges. This supports research that suggests resilience can be developed over time [22]. The differences between the two age groups might be due to youngsters developing resilience-boosting skills, resources, and support networks as they progress academically. Science is a difficult field that requires resilience. Students with excellent academic resilience tend to have a network of peers and mentors who help them manage stress and succeed academically. Personality traits like self-efficacy and self-esteem might affect academic resilience [23]. Self-confident students are more resilient and have the ability to manage stress and succeed academically. This study emphasizes the need to understand resilience's multifaceted traits and the many factors that can influence it in university students. Institutions should provide resilience treatments and support, especially for cognitively challenging students and younger students who may be more sensitive to mental health issues. We should develop ongoing monitoring systems to evaluate the effectiveness of implemented mental health interventions. This ensures that the programs remain relevant and impactful, adjusting based on feedback and changing needs.

Limitations

The study encountered several limitations related to validity and generalizability. The cross-sectional design, while useful for capturing a snapshot of relationships at a single point in time, limits the ability to assess changes in mental health and resilience over time. This design also precludes the determination of causal relationships between resilience and mental health markers, such as whether higher resilience reduces mental health issues or if lower mental health outcomes contribute to diminished resilience.

Additionally, the use of self-administered questionnaires presents potential biases. Participants may misinterpret questions, leading to inaccurate responses, or they may provide socially desirable answers rather than reflecting their true feelings and experiences. This introduces response bias, which can distort the results and compromise the study's validity. The sample size, while sufficient for exploratory purposes, was drawn from two faculties within a single university, limiting the generalizability of the findings to a broader student population. It is unclear whether the observed relationships between resilience and mental health apply equally across different faculties, universities, or geographical regions. Furthermore, the study only included university students, which restricts its applicability to other age groups or non-academic populations. To address these limitations, future research should consider employing longitudinal designs that track changes in mental health and resilience over time. This would allow for a clearer understanding of causal relationships and the long-term effects of resilience on mental health. Incorporating follow-up assessments can also provide insights into how resilience evolves and its impact on coping strategies.

## Conclusions

Bio-resources and food industry (FBIM) students of UniSZA have a higher rate of depression, anxiety, and stress than social science and humanities (FSSG) students of UniSZA. Resilience has a negative correlation with depression, anxiety, and stress, suggesting that it mitigates these mental health issues. We can conclude that science students have higher levels of depression, anxiety, and stress. This discrepancy suggests that science programs' rigorousness contributed to these students' mental health issues. The students had higher rates of depression, anxiety, and stress, highlighting the need for science student mental health services. Academic pressure, a complex curriculum, and high science expectations may contribute to mental health issues. Science students need specialized mental health programs and stress management resources. This study also found that male students are more depressed, anxious, and stressed than females. This study underlined the need for gender-specific mental health therapies to meet male students' unique challenges and coping techniques. Mental health issues are more prevalent.

## Appendices

Appendix A 

**Table 7:** Connor and Davidson Resilience Scale (CD-RISC) Connor, K. M., & Davidson, J. R. T. : Development of a new resilience scale: The Connor-Davidson resilience scale (CD-RISC). Depression and Anxiety. 2003, 18(2):76-82. 10.1002/da.10113 [6] 0 = Not true at all; 1 = Rarely true; 2 = Sometimes true; 3 = Often true; 4 = True nearly all the time

CD-RISC						
1	I am able to adapt when changes occur	0	1	2	3	4	
2	I have one close and secure relationship	0	1	2	3	4	
3	Sometimes fate or God helps me	0	1	2	3	4	
4	I can deal with whatever comes my way	0	1	2	3	4	
5	Past successes give me confidence	0	1	2	3	4	
6	I try to see the humorous side of things when I am faced with problems	0	1	2	3	4	
7	Having to cope with stress can make me stronger	0	1	2	3	4	
8	I tend to bounce back after illness, injury or other hardships	0	1	2	3	4	
9	I believe most things happen for a reason	0	1	2	3	4	
10	I make my best effort, no matter what	0	1	2	3	4	
11	I believe I can make my goals, even if there are obstacles	0	1	2	3	4	
12	Even when hopeless, I do not give up	0	1	2	3	4	
13	In times of stress, I know where to find help	0	1	2	3	4	
14	Under pressure, I stay focused and think clearly	0	1	2	3	4	
15	I prefer to take the lead in problem solving	0	1	2	3	4	
16	I am not easily discouraged by failure	0	1	2	3	4	
17	I think of myself as a strong person when dealing with life’s challenges and difficulties	0	1	2	3	4	
18	I make unpopular or difficult decisions	0	1	2	3	4	
19	I am able to handle unpleasant or painful feelings like sadness, fear and anger	0	1	2	3	4	
20	I have to act on a hunch	0	1	2	3	4	
21	I have strong sense of purpose in life	0	1	2	3	4	
22	I feel like I am in control	0	1	2	3	4	
23	I like the challenges	0	1	2	3	4	
24	I work to attain goals	0	1	2	3	4	
25	I take pride in my achievements	0	1	2	3	4	

Appendix B 

**Table 8:** Depression, Anxiety and Stress Scale-21 (DASS-21) Ng F, Trauer T, Dodd S, Callaly T, Campbell S, Berk M: The validity of the 21-item version of the Depression Anxiety Stress Scales as a routine clinical outcome measure. Acta Neuropsychiatr. 2007, 19:304-10. 10.1111/j.1601-5215.2007.00217.x [13] 0 = Did not apply to me at all 1 = Applied to me to some degree, or some of the time; 2 = Applied to me to a considerable degree or a good part of time; 3 = Applied to me very much or most of the time; a = anxiety; d= depression; s = stress

DASS-21					
1 (s)	I found it hard to wind down	0	1	2	3	
2 (a)	I was aware of dryness of my mouth	0	1	2	3	
3 (d)	I couldn’t seem to experience any positive feeling at all	0	1	2	3	
4 (a)	I experienced breathing difficulty (e.g., excessively rapid breathing, breathlessness in the absence of physical exertion)	0	1	2	3	
5 (d)	I found it difficult to work up the initiative to do things	0	1	2	3	
6 (s)	I tended to over-react to situations	0	1	2	3	
7 (a)	I experienced trembling (e.g., in the hands)	0	1	2	3	
8 (s)	I felt that I was using a lot of nervous energy	0	1	2	3	
9 (a)	I was worried about situations in which I might panic and make a fool of myself	0	1	2	3	
10 (d)	I felt that I had nothing to look forward to	0	1	2	3	
11 (s)	I found myself getting agitated	0	1	2	3	
12 (s)	I found it difficult to relax	0	1	2	3	
13 (d)	I felt down-hearted and blue	0	1	2	3	
14 (s)	I was intolerant of anything that kept me from getting on with what I was doing	0	1	2	3	
15 (a)	I felt I was close to panic	0	1	2	3	
16 (d)	I was unable to become enthusiastic about anything	0	1	2	3	
17 (d)	I felt I wasn’t worth much as a person	0	1	2	3	
18 (s)	I felt that I was rather touchy	0	1	2	3	
19 (a)	I was aware of the action of my heart in the absence of physical exertion (e.g. sense of heart rate increase, heart missing a beat)	0	1	2	3	
20 (a)	I felt scared without any good reason	0	1	2	3	
21 (d)	I felt that life was meaningless	0	1	2	3	
